# Topological traits of a cellular pattern versus growth rate anisotropy in radish roots

**DOI:** 10.1007/s00709-019-01362-6

**Published:** 2019-03-05

**Authors:** Anna Piekarska-Stachowiak, Joanna Szymanowska-Pułka, Izabela Potocka, Marcin Lipowczan

**Affiliations:** 1 Department of Biophysics and Morphogenesis of Plants, Faculty of Biology and Environmental Protection, University of Silesia, Katowice, Poland; 2 Laboratory of Microscopic Techniques, Faculty of Biology and Environmental Protection, University of Silesia, Katowice, Poland

**Keywords:** Cellular pattern, Growth rate anisotropy, Topology

## Abstract

The topology of a cellular pattern, which means the spatial arrangement of cells, directly corresponds with cell packing, which is crucial for tissue and organ functioning. The topological features of cells that are typically analyzed are the number of their neighbors and the cell area. To date, the objects of most topological studies have been the growing cells of the surface tissues of plant and animal organs. Some of these researches also provide verification of Lewis’s Law concerning the linear correlation between the number of neighboring cells and the cell area. Our aim was to analyze the cellular topology and applicability of Lewis’s Law to an anisotropically growing plant organ. The object of our study was the root apex of radish. Based on the tensor description of plant organ growth, we specified the level of anisotropy in specific zones (the root proper, the columella of the cap and the lateral parts of the cap) and in specific types of both external (epidermis) and internal tissues (stele and ground tissue) of the apex. The strongest anisotropy occurred in the root proper, while both zones of the cap showed an intermediate level of anisotropy of growth. Some differences in the topology of the cellular pattern in the zones were also detected; in the root proper, six-sided cells predominated, while in the root cap columella and in the lateral parts of the cap, most cells had five neighbors. The correlation coefficient *r*_L_ between the number of neighboring cells and the cell area was high in the apex as a whole as well as in all of the zones except the root proper and in all of the tissue types except the ground tissue. In general, Lewis’s Law was fulfilled in the anisotropically growing radish root apex. However, the level of the applicability (*r*_L_ value) of Lewis’s Law was negatively correlated with the level of the anisotropy of growth, which may suggest that in plant organs in the regions of anisotropic growth, the number of neighboring cells is less dependent on the cell size.

**Electronic supplementary material:**

The online version of this article (10.1007/s00709-019-01362-6) contains supplementary material, which is available to authorized users.

## Introduction

Plant organs grow symplastically, that is to say that the cells within the organs grow in a highly coordinated manner (Priestley 1930; Erickson 1986). This coordination means that during growth, cells do not slip or slide with respect to each other, and that an organ maintains its integrity over time. The highly organized growth of plant organs is strictly connected with the topology of their cellular pattern. In the axial sections of the plant shoot and root apices, the cells are densely packed and arranged in continuous files and therefore the cell wall network can be described by two families of continuous and mutually orthogonal lines—periclines and anticlines—which are parallel and perpendicular to the surface of an organ, respectively (Sachs 1879, 1887). This pattern of anti- and periclines is maintained during the steady growth of an organ (Sachs 1887; Hejnowicz 1982).

The root apex—an organ responsible for development of the whole root—consists of the root proper (rp) and the root cap. In most angiosperms and in some gymnosperms, growth of the apex is governed by quiescent center (QC) localized on the pole of the root proper. The QC is surrounded by initials that are organized in cell tiers from which the files of the cells originate. In *Arabidopsis* and radish, where three such tiers occur, the most distal tier gives rise to the root cap and epidermis (E), the middle tier produces the ground tissue (G) comprising the cortex and endodermis, and the innermost tier generates the pericycle and vascular tissues that constitute the stele (S) (Kadej 1970; Dolan et al. 1993). The cells of the root proper do not grow onto the side of the cap and that is why this type of root apex organization is termed closed (Clowes 1981). The cells in the QC grow very slowly or do not grow at all. In the root proper, cell growth takes place toward the base of the root, in the columella toward the tip and in the lateral regions of the cap toward the flanks.

**Fig. 1:**
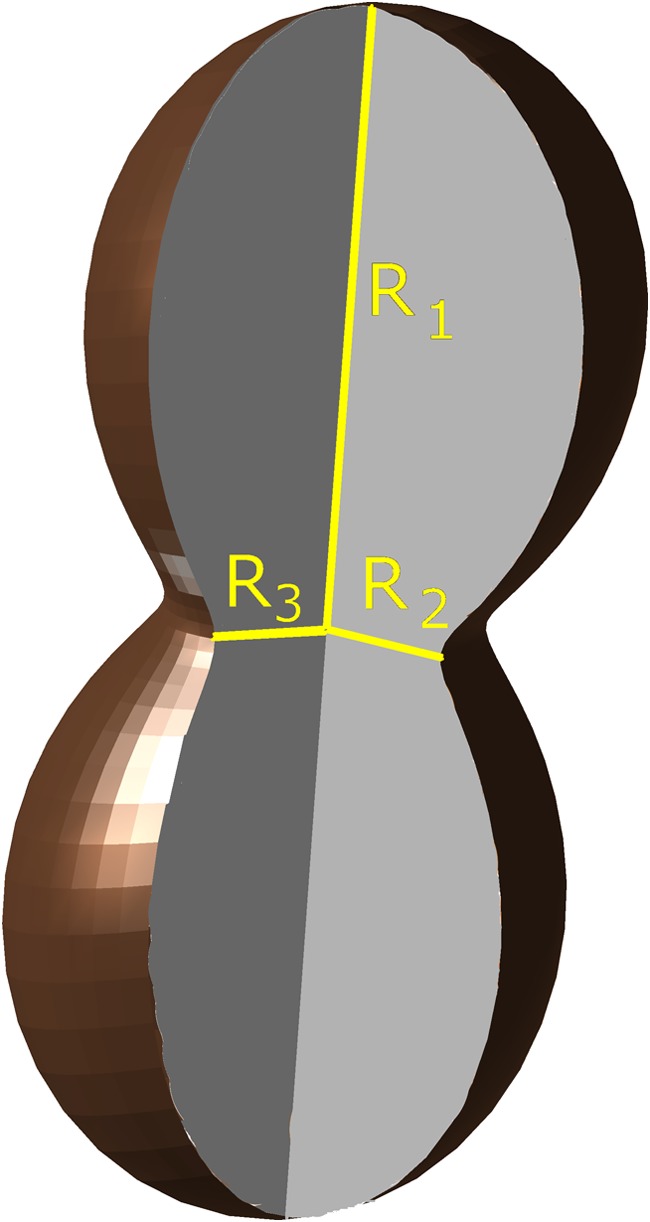
Graphical representation of a single indicatrix. The length of a section from the center point to the surface of the indicatrix indicates the value of the growth rate in the direction in which the section is oriented. The yellow sections indicate the values of the growth rates (*R*_1_, *R*_2_, and *R*_3_) in the three principal directions: maximal (1), minimal (2), and saddle type (3), respectively

To describe the symplastic growth of a plant organ, the growth tensor (GT) (Hejnowicz and Romberger 1984) can be applied, which enables the growth rates to be calculated at every point of the organ and in every direction, thus providing a quantitative representation of the growth distribution (or growth field) in an organ. Growth rates (*R*), which are calculated at a selected point of an organ in many directions, can be graphically represented by an indicatrix, which is the 3D closed surface whose central position is taken by the point (Fig. 1). The distance from the point to the surface in a given direction is determined by the value of *R* in this direction. That is why the shape of the indicatrix depends on the character of growth at that point (Nakielski and Lipowczan 2013; Szymanowska-Pułka and Lipowczan 2014). If the growth at a point is isotropic, the indicatrix is a sphere, while in the case of anisotropic growth, the indicatrix is elongated along the direction of the strongest growth (Fig. 1). The growth of most plant organs is anisotropic. In such a case, three mutually orthogonal principal directions of growth (PDGs) (Hejnowicz and Romberger 1984) can be distinguished at every point of the organ. In two of these directions, namely, maximal (1) and minimal (2), the growth is extreme. The third direction is perpendicular to the plane that is formed by the two and is called the saddle (3) direction (Szymanowska-Pułka and Nakielski 2010; Nakielski and Lipowczan 2013). In Fig. 1, the PDGs are indicated by the yellow lines along which the growth rates reach the values *R*_1_, *R*_2_, and *R*_3_, respectively. The principal directions are arranged in orthogonal trajectories that correspond with the above-mentioned pattern of anti- and periclines, and that is why these lines were postulated as representing the PDG trajectories (Hejnowicz 1984, 1989). In a steadily growing plant organ whose shape does not change over time, the pattern of the PDG trajectories remains steady (Hejnowicz 1984; Szymanowska-Pułka 2007; Szymanowska-Pułka and Nakielski 2010). According to Hejnowicz’s hypothesis (1984, 1989), the orientation of a newly inserted cell wall during cell division is determined by the plane that is formed by two of the PDGs. That is why in the roots (Nakielski 1987, 2008) and other plant organs (Dumais and Kwiatkowska 2002; Kwiatkowska 2004), the pattern of the PDG trajectories can be observed in the cell wall system.

Every individual plant organ has its own growth field. The growth distribution within an organ may be graphically represented by a map of the indicatrices that are determined at selected points (Hejnowicz and Karczewski 1993). Based on anatomical observations and the GT definition (Hejnowicz and Romberger 1984), this type of field was determined and applied to simulate the growth of the shoot (Nakielski 2000) and root apices (Hejnowicz and Karczewski 1993; Nakielski and Lipowczan 2012; Szymanowska-Pułka et al. 2012; Piekarska-Stachowiak and Nakielski 2013) in various plant species. In the simulations, the plant organ being considered is described in 2D and its cells are represented by polygons. Nakielski (2008) specified such a field in the radish root apex (Supplementary Material, Eq. 1), on the basis of which a map of the growth rates in this organ was constructed (Supplementary Material, Fig. 1). This field consists of four zones (in Supplementary Material, Fig. 1 separated by green lines) that refer to the four zones of the root apex: QC, rp, cc, and lc.

While coordinated cell proliferation is a consequence of cell divisions in the planes that are determined by the PDGs, the regularity in the shape of cells and in the cellular pattern may result either from the former or from the more or less steady number of neighboring cells in specific tissue types. The latter is an element of the topology of a cellular pattern. The average number of neighbors of a cell in the 2D view was found to be approximately six (see the review paper by Gibson and Gibson 2009) due to the three-cell junctions that are prevalent (Gibson et al. 2006; Nakielski 2008; Li et al. 2012). As early as in 1926, Lewis showed that the cucumber epidermal cells that were observed in 2D were mostly six-sided, which means that they might be described as hexagons (Lewis 1926). The predominance of six-sided cells has also been reported by other authors in various plant (Lewis 1928; Korn and Spalding 1973; Mombach et al. 1990; Pina and Fortes 1996; Sahlin et al. 2009; Carter et al. 2017) and animal species (Zallen and Zallen 2004; Classen et al. 2005; Gibson et al. 2006; Nagpal et al. 2008; Sandersius et al. 2011; Xu et al. 2017). The above-mentioned results concern 2D description of single-type cells.

Cells manifest a tendency to remain in a state of equilibrium during organ growth and the associated divisions of cells (Lewis 1928; Rivier et al. 1995; Gibson and Gibson 2009). This enables the steady average size of cells within the growing regions to be maintained, which was also confirmed in modeling and simulation of the plant organ growth (Hejnowicz and Karczewski 1993; Szymanowska-Pułka et al. 2012; Piekarska-Stachowiak and Nakielski 2013). Moreover, the arrangement of cells in growing regions seems to be to fill the topological space optimally (Rivier et al. 1995; Gibson and Gibson 2009). It has been shown that in the case of isotropic cell growth, there is a linear correlation between the average size and the shapes of cells (Lewis 1928). In other words, the larger the cell area is, the larger the number of the sides of the polygon that represents the cell in a 2D view is. This dependency, which has been designated as Lewis’s Law, can be observed in cells of both plant (Mombach et al. 1990; Pina and Fortes 1996; Sahlin and Jonsson 2010) and animal (Nagpal et al. 2008) organs in nature. In most cases, the Law was verified in the cells of surface tissues, such as the epidermis of plant organs (Lewis 1928; Mombach et al. 1990; Sahlin et al. 2009; Sahlin and Jonsson 2010; Kim et al. 2014) and epithelium of animal organs (Nagpal et al. 2008). The only example of the verification of Lewis’s Law in internally localized cells is the Pina and Fortes (1996) study on cork in *Quercus suber*. However, whether it concerns the external or internal tissues, studies on the application of Lewis’s Law are always conducted on cells of a single type. To date, no study has been run on applicability of Lewis’s Law in tissues considered as 3D structures. One of the few examples of description of cell geometry in 3D comes from work on tomato roots (Duffy 1951); however, the research was limited to the number of cell sides while neither the cell area nor the cell volume were analyzed and consequently no possible correlation between the cell size and the number of sides was verified.

Topological data provide information about cell packing, which is very important for tissue and organ functioning (Classen et al. 2005). They also complement the data from anatomical research. Yet, the above-presented short literature review shows that there is a lack of research concerning both the topology and the applicability of Lewis’s Law to the case of the cells in the different tissues/zones of the same organ. From previous studies (Hejnowicz and Karczewski 1993; Piekarska-Stachowiak and Nakielski 2013), we know that the different tissues and zones of a plant organ grow at different rates. That is why we were interested in performing a detailed analysis of the anisotropy of plant organ growth as well as of the topology of the cells that form the specific zones and tissues of the radish root apex in its axial section. Our aim was to estimate the strength of the anisotropy of the growth rates in specific zones of the apex and to examine the applicability of Lewis’s Law to this plant organ. Another specific objective of this study was to investigate whether the level of anisotropy affects the correlation between the number of cell neighbors and the cell area. The results provide exclusive knowledge about the growth of the radish root apex as well as a detailed description of the cell topology in the different zones and tissues of the meristematic region of this organ.

## Methods

### Plant material

Seeds of radish (*Raphanus sativus* L. cv. Mila) were soaked overnight and germinated in vertically oriented rolls of moist filter paper for 3 days at room temperature. For the anatomical observations, 2–3-mm terminal segments of the primary roots were excised and fixed in 2.5% glutaraldehyde in a 0.05 M sodium phosphate buffer (pH 7.0) for 24 h, washed three times in the buffer, dehydrated through an ethanol series and propylene oxide, and then embedded in Epon. The samples were sectioned into longitudinal sections (2.5 μm thick) using a Tesla BS 490A ultramicrotome. Some of the root tips were also embedded in low-melting polyester wax (Steedman’s wax) as described by Vitha et al. (2000) and cut to a thickness of 7 μm using a HYRAX M 40 electronic rotary microtome (Carl Zeiss MicroImaging GmbH). The sections were stained with a periodic acid-Schiff (PAS) reaction (O’Brien and McCully 1981) and observed using an Olympus BX41 microscope equipped with an Olympus XC50 camera. Images of the axial sections of 12 root apices were selected and analyzed.

### Data analysis

The cell pattern of the axial sections of the root apices was redrawn carefully in order to obtain the set of polygons that represented the cells. The cells of the non-growing QC region were not taken into consideration in the analysis. Cells whose outline was not clear, cells that underwent sloughing, and the most external cells whose growth had ended (Barlow 2003) were also omitted from the analysis. The anisotropy coefficient was calculated in the geometrical center of each of the analyzed cells according to the following formula: $A=\left|\frac{R_1-{R}_2}{R_1+{R}_2}\right|$ (Dumais and Kwiatkowska 2002; Dumais et al. 2004), where *R*_1_ and *R*_2_ are growth rates in the two principal directions of growth that are visible in the plane of the axial section of the root apex. *R*_1_ and *R*_2_ were determined based on the growth tensor field that was dedicated to the radish root apex (Nakielski 2008; see also Supplementary Material, Eq. 1 in Kucypera et al. 2017). Notice that the anisotropy coefficient takes the value from the range of 0 to 1, where 0 refers to the isotropic growth and 1 refers to the extreme anisotropic growth (growth in one direction). The area and the number of cell sides (= the number of neighboring cells = the number of cell vertices) in the polygon meshwork were determined for all of the cells that were clearly visible in the sections of the analyzed root apices. The calculations of the anisotropy coefficient *A* and the measurements of the number of cell sides and cell areas (in μm^2^) were performed using the software that was originally elaborated by the authors of this current paper in the MATLAB environment, Matworks.

### Statistical analysis

The chi-squared goodness-of-fit test (*p* < 0.05) was used to examine the normality of the distribution of the anisotropy coefficient, the cell area, and the number of cell sides of the 12 root apices. The cell area strongly differed in the various regions of the root apex, which caused a large standard deviation of the mean. For this reason, to present Lewis’s Law in a form of correlation equation, these data were standardized (separately for each root apex) according to the following formula: *z* = (*x* − *m*)/*s*, where *z* is the standardized value of the cell area, *x* is the actual value of the cell area, *m* is the mean value of the data for a given root apex, and *s* is the standard deviation of the mean. Standardization of data is a widely applied method in this type of study (Sahlin and Jonsson 2010; Abera et al. 2014). In most cases, the characteristics did not have a normal distribution and that is why a significance of statistical differences between the 12 root apices based on a comparison of the medians of the anisotropy coefficient, the cell area, and the number of cell sides (*n*) was estimated using the nonparametric Kruskal-Wallis statistics. The Pearson correlation coefficient was calculated and the linear regression was used to test Lewis’s Law, which is the potential linear relationship (*r*_L_) between the cell area and the number of cell sides. The same statistical method was used to determine the correlation (*r*_LA_) between the anisotropy coefficient *A* and coefficient *r*_L_. All of the statistical calculations were performed in Statistica 12, StatSoft Inc.

## Results

**Fig. 2:**
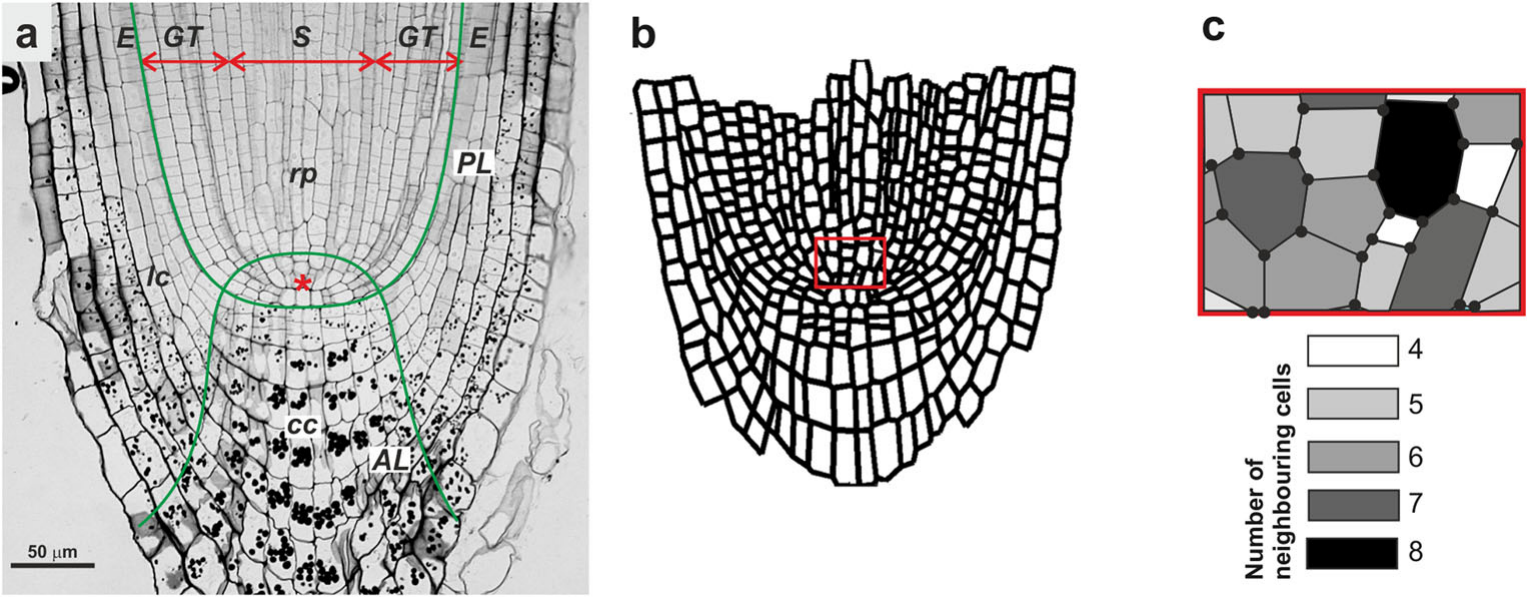
Axial view of a representative root apex in radish (**a**), its cell pattern that was redrawn as a set of polygons for further analysis (**b**, see the “Methods” section), and a fragment of the cell pattern framed in **b** with gray scale code indicating the number of neighboring cells (**c**). The cell walls form a regular pattern of periclines and anticlines. In **a**, the pericline (PL) that indicates the root proper (rp) and the root cap border and the anticline (AL) that indicates the proximal range of the QC (asterisk) are distinguished (green). The root cap consists of the columella (cc) and lateral parts (lc). The cell files that form tissue types are indicated as follows: *E* epidermis, *G* ground tissue (cortex and endodermis), *S* stele (pericycle and vascular tissue)

In Fig. 2, the axial section of the root apex in radish with its particular zones and tissues is shown. In this view, the regular pattern of periclines and anticlines that is formed by the cell walls is visible. A small QC is localized on the pole of the root proper. One of the periclines (Fig. 2a, PL) refers to the root proper/root cap border while one of the anticlines (Fig. 2a, AL) shows the proximal limits of the QC and separates the central part of the root cap (root cap columella, cc) from the lateral parts of the cap (lc). Tangents at any point to the peri- and anticlines determine the PDGs at the point. The cell pattern of the same root apex represented in a form of a set of polygons is shown in Fig. 2b. The slowly growing cells of QC and the oldest external cells were not taken into consideration (compare Fig. 2a and Fig. 2b). An enlarged fragment of the root apex from Fig. 2b is shown in Fig. 2c with gray scale-coded cells of various numbers of neighbors. In this meristematic area, differences in both the cell size and number of cell sides result from frequent cell divisions.

From the 12 root apices in their axial view, 4944 cells were analyzed for their anisotropy coefficient, area, and number of neighbors.

### Anisotropy coefficient

**Fig. 3:**
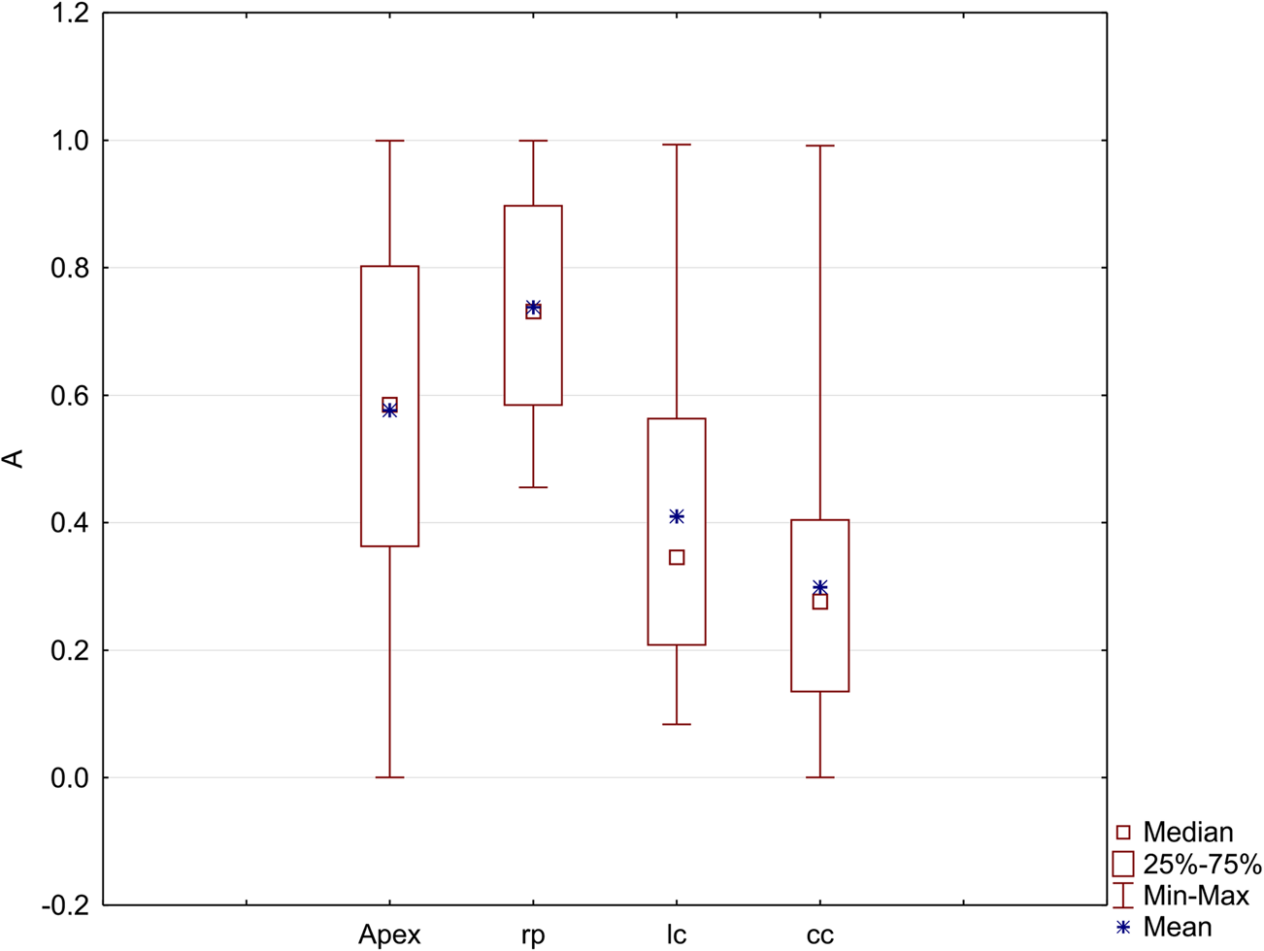
Medians and quartiles of the variable *A* (anisotropy coefficient) in the apex and in specific zones of the 12 roots (rp, root proper; lc, lateral parts of the root cap; cc, columella of the root cap). The lack of significant differences between the medians within the zones and in the entire apex for the 12 roots was proven using Kruskal-Wallis statistics (*p* < 0.05). Mean values of *A* in particular zones are indicated to compare with medians

The anisotropy coefficient (*A*) was calculated for each cell of the root apices. The results, which were organized into four groups that refer to the entire apex and to specific zones (rp, lc, cc), are presented in the form of box-and-whisker plot (Fig. 3), which shows the characteristics of the data coming from the 12 analyzed roots. The chi-squared goodness-of-fit test (*p* < 0.05) that was used indicated a lack of a normal distribution of variable *A* for most of the roots. For this reason, the Kruskal-Wallis tests were applied separately to each zone and to the entire apex in order to determine the possible differences between the analyzed roots. The tests, however, revealed no statistical differences between the roots based on a comparison of the anisotropy coefficient (*p* = 0.07, *p* = 0.08, *p* = 0.11, *p* = 0.09, for the apex, rp, lc, and cc, respectively).

**Fig. 4:**
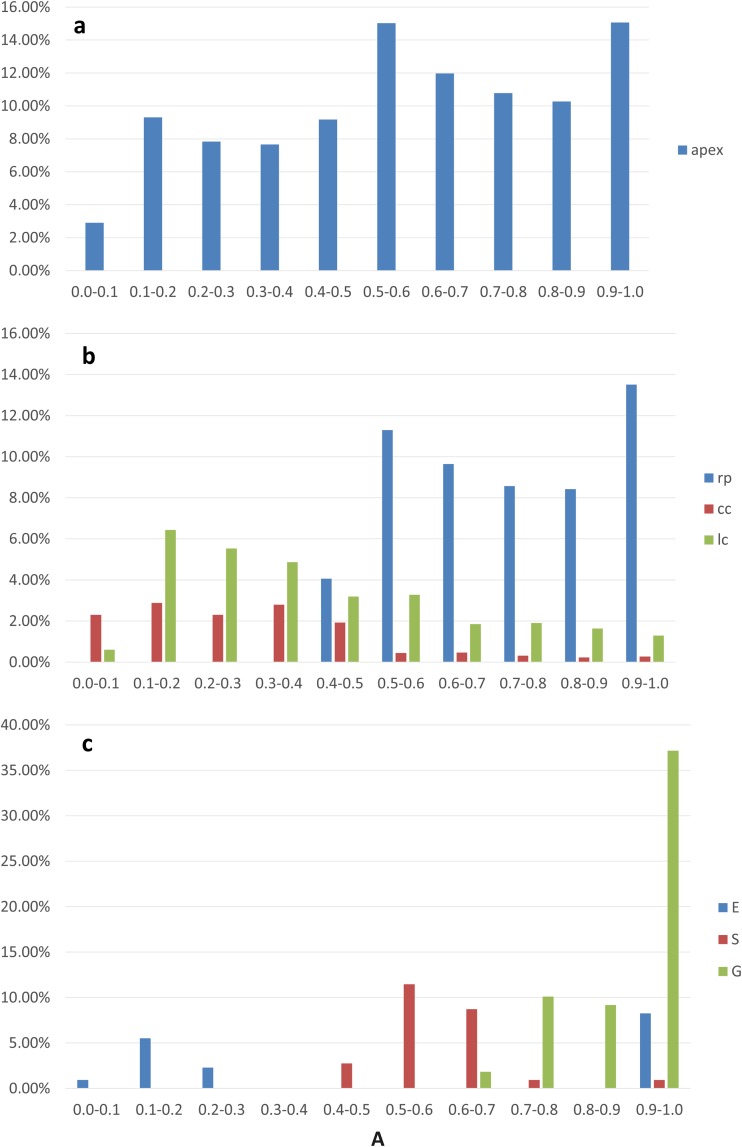
Variety in the growth rate anisotropy *A* in the root apex (**a**) and in the considered root zones (**b**) and tissues (**c**). The bars in specific ranges of *A* in **a** are sums of the bars of the same ranges in **b**. The data in **a** and **b** are from the 12 analyzed roots, data in **c** are from the representative apex that is shown in Fig. 2a. apex, the entire apex; rp, root proper; cc, root cap columella; lc, lateral parts of the cap; E, epidermis; S, stele; G, ground tissue

The data for all of the analyzed apices were taken into consideration to show the distribution of the anisotropy coefficient in the root apex as a whole (Fig. 4a) and in its zones (Fig. 4b). The frequencies referring to the ranges of the value of *A* in Fig. 4a are the sums of the frequencies in the same ranges in Fig. 4b, which means that the frequencies in the zones contribute to the frequencies in the entire apex. In the apex (Fig. 4a), cells with an intermediate (0.5–0.6) or large (0.9–1.0) value of *A* were most numerous (about 15% of the population for both). Only about 3% of the cells showed small anisotropy of growth (0.0–0.1). The histogram in Fig. 4b shows the characteristic polarization of the *A* value in the zones, namely, strong anisotropic growth was present in the root proper, while in the columella of the cap and in the lateral regions of the cap, lower values of *A* predominated although the coefficient had values from the whole range of 0 to 1 in both zones.

As was mentioned earlier, no statistical differences were detected between the anisotropy coefficients of the root apices and that is why the representative root apex (shown in Fig. 2a) was taken into consideration to analyze the distribution of the anisotropy coefficient in the tissues (Fig. 4c). In epidermal cells, growth is either close to isotropic (*A* in the range 0–0.3) or extremely anisotropic (0.9–1.0). In the stele, *A* reached intermediate values (mostly 0.4–0.7), while in the ground tissue, the cells grew extremely anisotropically: the most frequent were the cells for which *A* was close to 1. Taken together, the results presented above prove an anisotropic character of the growth of the root apex in radish.

### The cell area and the number of cell sides

**Fig. 5:**
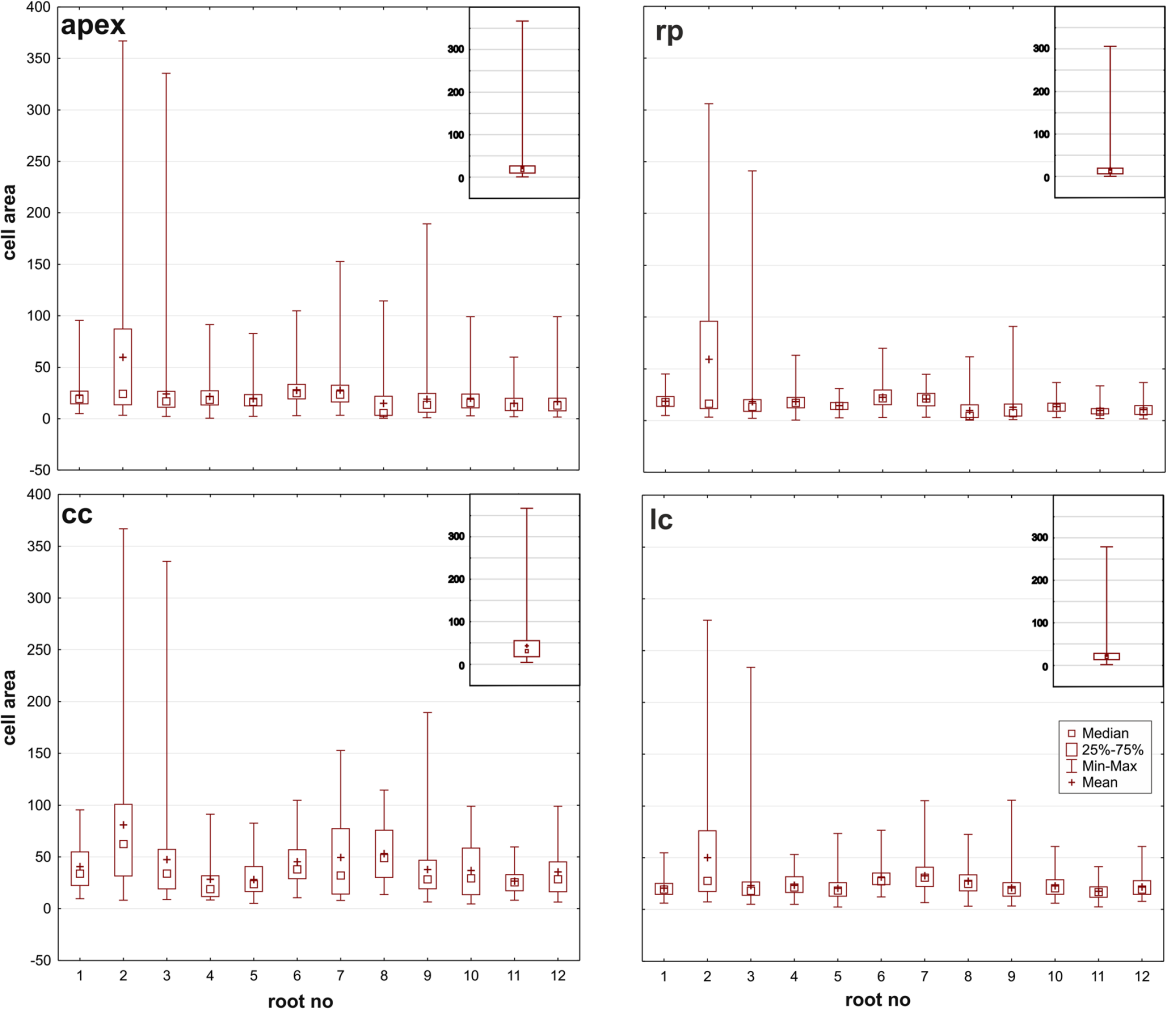
Structures of the variable “cell area” [μm^2^] in the analyzed root apices (apex) and in their specific zones (rp, root proper; lc, lateral parts of the root cap; cc, columella of the root cap). The Kruskal-Wallis statistical test (*p* < 0.05) revealed a lack of significant differences between the medians within the zones and in the entire apex for the 12 roots. Mean values of cell area are indicated to compare with medians. Insets present characteristics of the cell area in the apex and the zones taken together from all the analyzed roots

**Fig. 6:**
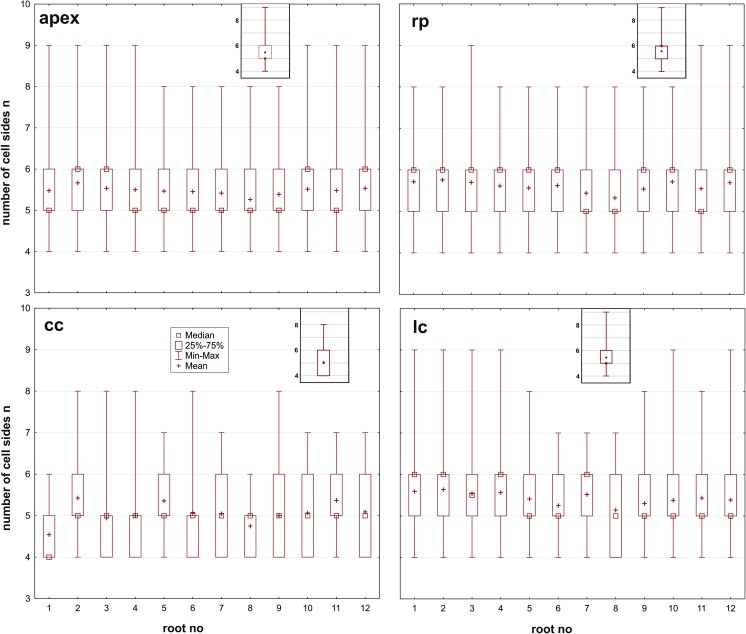
Structures of the variable “number of cell sides” *n* in the analyzed root apices (apex) and in their specific zones (rp, root proper; lc, lateral parts of the root cap; cc, columella of the root cap). The Kruskal-Wallis statistical test (*p* < 0.05) revealed a lack of significant differences between the medians within the zones and in the entire apex for the 12 roots. Mean values of number of cell sides are indicated to compare with medians. Insets present characteristics of the number of cell sides in the apex and the zones taken together from all the analyzed roots

Next, the cell area and the number of cell sides of each analyzed cell of the root apices were determined. As in the case of the asymmetry coefficient, the data were organized into four groups that refer to the entire apex and to the three zones (rp, cc, lc). The characteristics of the data are presented in Fig. 5 (cell area) and Fig. 6 (cell sides). The distributions of both variables were far from symmetrical. In some cases, the distribution was extremely skewed, for example, the cell area in all of the zones of roots apices 2, 3, and 9 (Fig. 5) as well as the number of cell sides in the root cap columella of all of the roots (Fig. 6). Root apices 2 and 3 had a wide range of cell area compared to the other apices (Fig. 5). The medians of the number of cell sides were six in the root proper and five in the root cap columella, in the lateral regions of the cap, and in the entire apex (Fig. 6). Both variables demonstrated a lack of the normal distribution (the chi-squared goodness-of-fit test, *p* < 0.05). No statistically significant differences were found between the analyzed roots when the medians were compared (Kruskal-Wallis statistics, *p* < 0.05) in either the cell area (*p* = 0.85, *p* = 0.98, *p* = 0.99, *p* = 0.45, for the apex, rp, cc, and lc, respectively) or in the number of cell sides (*p* = 0.67, *p* = 0.14, *p* = 0.83, *p* = 0.19, for the apex, rp, cc, and lc, respectively).

**Fig. 7:**
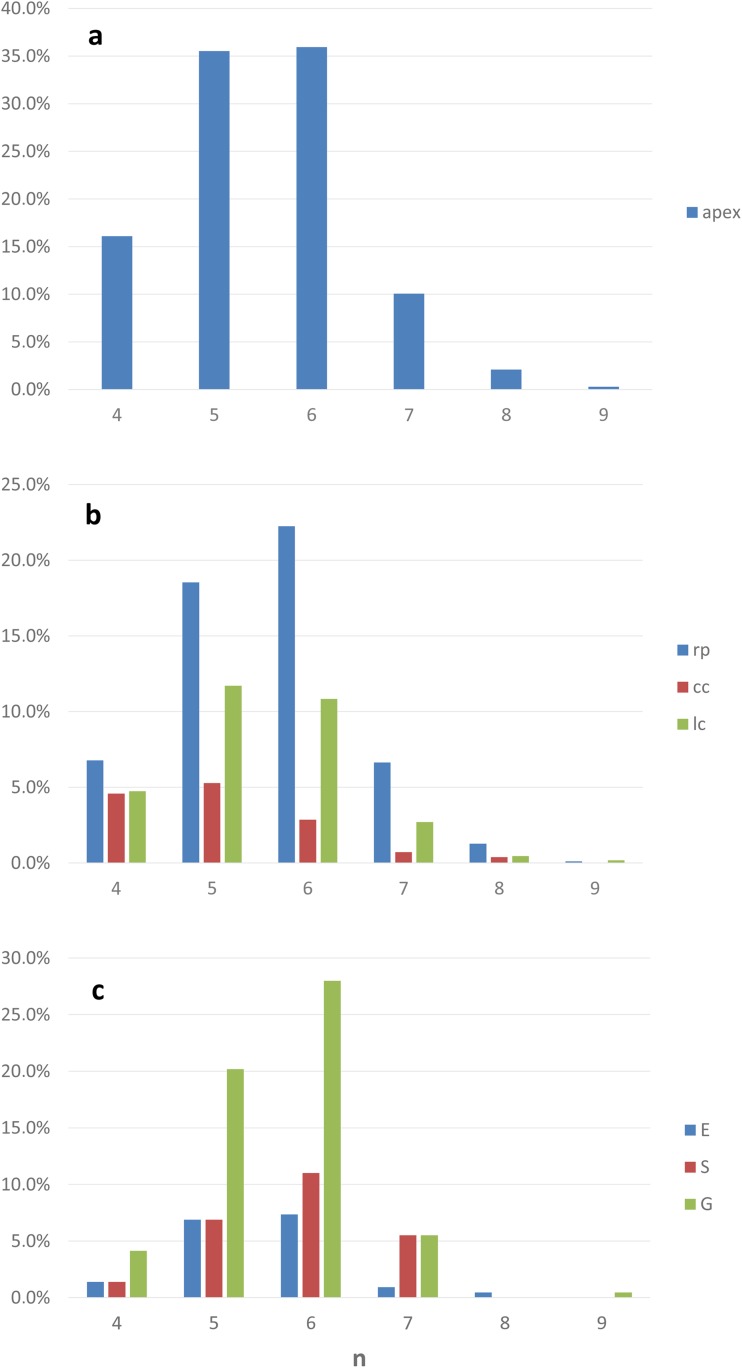
Distribution of the number of cell sides in the entire apex (**a**), in the considered zones (**b**), and in specific tissues of the root proper (**c**). Data in **a** and **b** are from the 12 analyzed roots, and data in **c** are from the representative apex that is shown in Fig. 2a. The bars that refer to the specific numbers of cell sides in **a** are the sums of the bars that refer to the same numbers in **b**. apex, the entire apex without the QC; rp, root proper; cc, root cap columella; lc, lateral parts of the cap; E, epidermis; S, stele; G, ground tissue

In Fig. 7, the distribution of the number of cell sides in the entire apex (Fig. 7a) and in specific zones (Fig. 7b) from the analyzed root apices is shown. The frequencies that refer to the specific number of cell sides in Fig. 7a are the sums of the frequencies that refer to the same numbers in Fig. 7b, which means that the frequencies in the zones contributed to the frequencies in the entire apex. In the apex, six-sided and five-sided cells (both over 35%) were observed most frequently, which means that the distribution of the cell number was binomial with modes *M*_1_ = 5 and *M*_2_ = 6; other polygons were less frequent (Fig. 7a). A similar distribution occurred in the root proper with 22% hexagons and 19% pentagons and other less numerous classes (Fig. 7b). Interestingly, the number of cell sides was a bit differently distributed in the root cap columella and in the lateral root cap. In the columella, five-sided (over 5%) and four-sided cells were the most frequent (almost 5%), while hexagons and other polygons were in the minority (Fig. 7b). In the lateral regions of the cap, pentagons (12%) and hexagons (11%) predominated, while other classes were in the minority.

To analyze the distribution of the number of cell sides in the tissues (Fig. 7c), the data from the representative root apex (Fig. 2a) were taken into consideration because (as shown above) no statistical differences were found between the root apices based on a comparison of this variable. In all of the tissues, the hexagon cells were dominant (7%, 11%, and 28% for the epidermis, stele, and ground tissue, respectively). The pentagons were the second most numerous group (7% for the epidermis and stele and 20% for the ground tissue). In the stele and ground tissue, heptagons were relatively numerous (more than 5%), while in epidermis, they were rather rare (Fig. 7c).

### Lewis’s Law verification

**Fig. 8:**
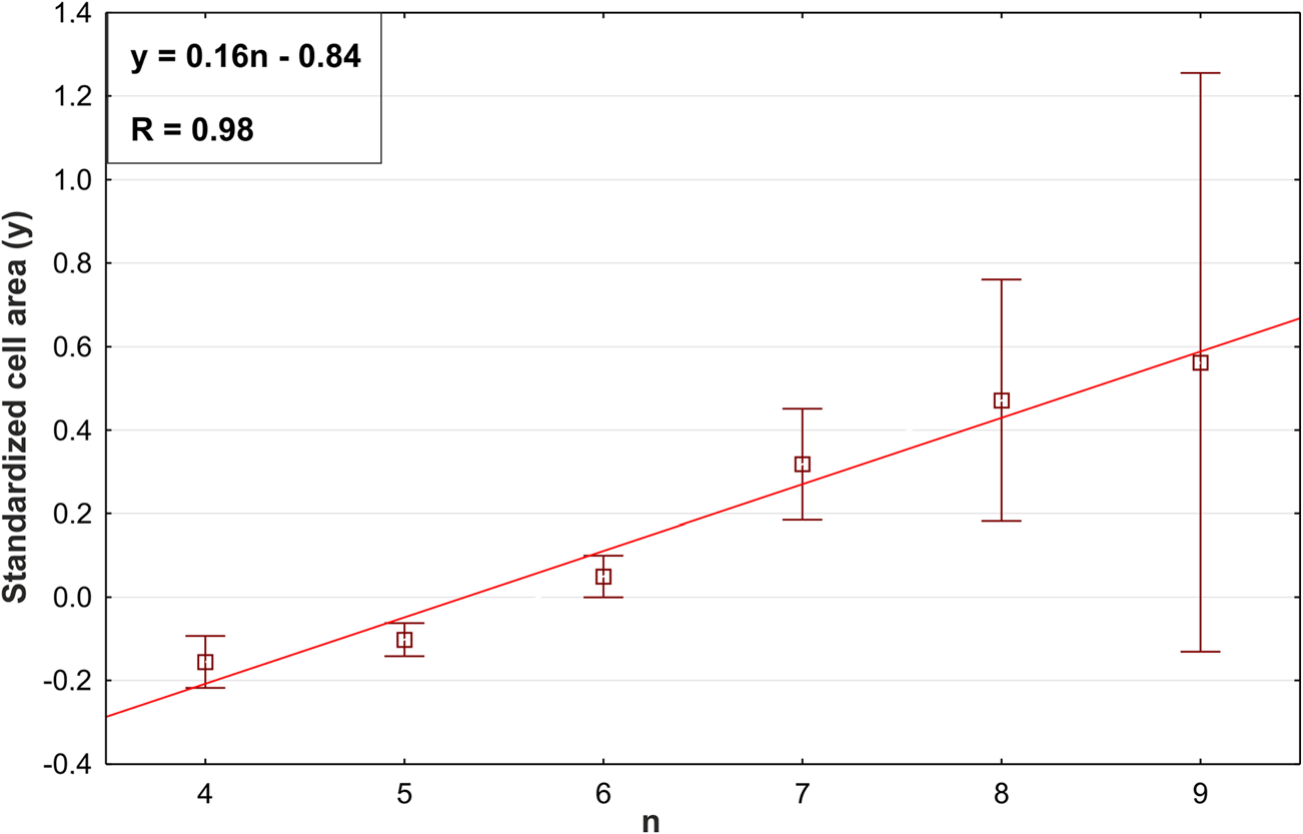
The linear relationship between the number of cell sides *n* and the standardized area *y* for the cells of the entire root apex. The data are means ± standard deviation. In the upper left corner, the equation *y*(*n*) and the coefficient of determination *R* = *r*_L_^2^ are given

Eventually, the relation between the standardized area of the cells and the number of the sides of the cells for the analyzed root apices taken together was tested using Pearson correlation statistics. Figure 8 presents the linear dependence of the two variables. The high correlation coefficient (*r*_L_ = 0.99) suggests that Lewis’s Law was fulfilled in the cells of the entire radish root apex. However, we decided to verify Lewis’s Law in specific zones of the root apex (the root proper, the columella of the cap, the lateral parts of the cap) and in specific tissues (the epidermis, stele, ground tissue) due to the differences in the growth rate anisotropy (Fig. 4).

In Table 1, the correlation coefficient (Lewis’s Law coefficient *r*_L_) between the standardized area of the cells (*y*) and the number of sides of the cells (*n*) in a representative root apex (Fig. 2), in its specific zones and in the analyzed tissues and the corresponding regression equations, is presented. The correlation between the variables was high in the entire apex. In the root proper, the *r*_L_ was also relatively high; however, in the tissues of that zone (the stele and ground tissue), its value ranged from 0.32 (G) to 0.99 (S). In the two other zones (lateral cap and columella), the correlation was very high. Thus, although Lewis’s Law was fulfilled in all of the zones of the apex, the correlation was rather weak in the ground tissue.

**Table 1:** The Pearson correlation coefficient and the regression equations that show the linear dependence between the standardized cell area and the number of cell sides in a representative root apex, its zones, and the analyzed tissues. In the regression equations, *y* is the standardized cell area and *n* is the number of neighboring cells

	Whole apex	Zones			Tissues		
		Root proper	Collumella	Lateral cap	Epidermis	Stele	Ground tissue
*r*_L_	0.99	0.60	0.94	0.99	0.79	0.99	0.32
Regression equation *y*	0.16*n* – 0.84	0.52*n* – 2.66	0.20*n* – 1.01	0.33*n* – 1.79	0.65*n* – 3.43	0.67*n* – 3.97	0.09*n* – 0.58

**Fig. 9:**
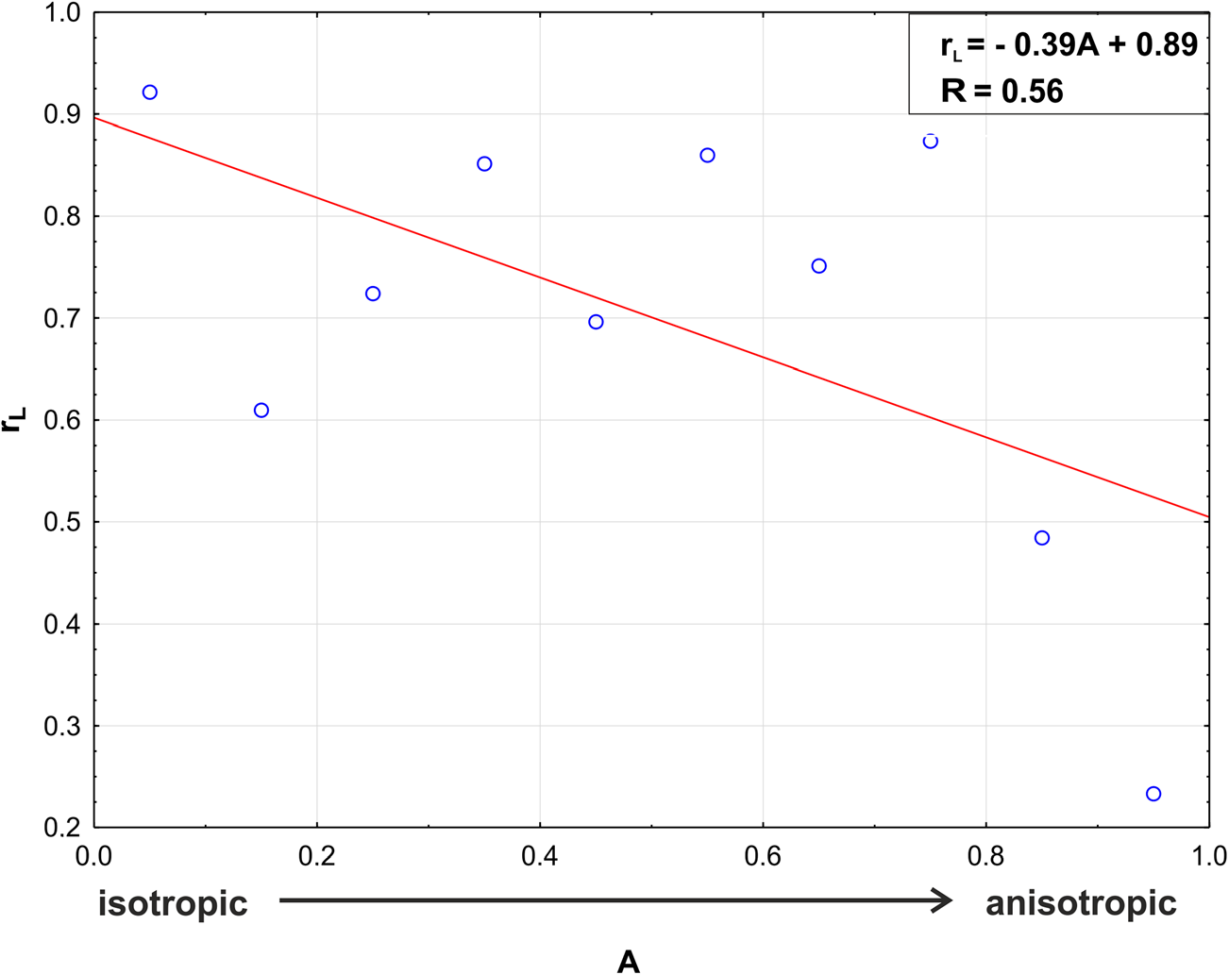
A linear negative dependence between the Lewis’s Law coefficient (= Pearson correlation coefficient *r*_L_) and the anisotropy coefficient (*A*); data from all the examined root apices were taken into consideration. In the right upper corner, the regression equation *r*_L_ (*A*) and coefficient of determination *R* are given

When the data from Table 1 was compared with the anisotropy distribution in the apex (Fig. 4), it suggested that there might be some relationship between the value of the *r*_L_ coefficient and the value of anisotropy coefficient *A*. The Pearson statistics proved a negative correlation (Fig. 9, *r* = − 0.56), which means that Lewis’s Law coefficient *r*_L_ decreases with an increasing anisotropy coefficient *A*. This suggests that Lewis’s Law is poorly applicable in regions of strong anisotropic cell growth.

## Discussion

### Growth of the root apex

The growth of a typical plant root concerns its elongation, while its transverse dimensions are more or less preserved (Baluška et al. 1990). Although this description suggests a simple case of extreme anisotropic growth, a detailed analysis of the distribution of the growth rates in root apices (Supplementary Material, Fig. 1; Nakielski 2008; Nakielski and Lipowczan 2013) as well as a quantitative analysis of the growth anisotropy (Figs. 3 and 4) reveal that the growth in the apical part of the root is more complex. In radish, the most anisotropic growth (the highest values of anisotropy coefficient A) was found in the region above QC, namely, in the root proper, where the cells elongated rapidly. Growth that is closest to isotropic (the lowest values of *A*) occurred below the QC in the root cap columella and in the lateral regions of the cap (see Fig. 3 and Supplementary Material, Fig. 1). In the entire apex, the average value of anisotropy coefficient *A* was slightly less than 0.6, which indicates that there is a level of intermediate asymmetry level. Interestingly, in the side regions of the apex where the oldest cells undergo sloughing, negative growth occurs (Supplementary Material, Fig. 1). These regions, however, were not taken into consideration in our analysis of the data that was used to verify the applicability of Lewis’s Law (compare Fig. 2a and Fig. 2b).

### Cell pattern topology

In simulations of the growth of plant organs (Nakielski 2008; Szymanowska-Pułka and Nakielski 2010; Szymanowska-Pułka et al. 2012; Lipowczan et al. 2013; Lipowczan and Piekarska-Stachowiak 2014; Kucypera et al. 2017) that are based on the growth tensor field (Hejnowicz and Romberger 1984), the stability and realistic appearance of the cell pattern depend heavily on the orientation of the division walls along the PDGs. Some recent modeling studies (Patel et al. 2009; Sahlin and Jonsson 2010; Li et al. 2012; Sahlin et al. 2009) have clearly shown that the orientation of cell division also affects the cell topology, especially the number of cell sides versus the cell area. Analyses of the geometry and topology in real isotropically growing plant (Lewis 1928; Korn and Spalding 1973; Pina and Fortes 1996) and animal (Gibson et al. 2006) organs have shown that in a 2D view of an organ six-sided cells are predominant, five- and seven-sided cells are less frequent (with a slight predominance of five-sided cells), while four- and three-sided as well as eight-, nine-, and ten-sided cells rarely occur. In other words, the distribution of a variable “number of cell sides” or an equivalent “number of neighboring cells” is more or less symmetrical with a mode = 6. Although in the case of the radish root apex, the most frequent cells were six-sided cells, the distribution of this variable was generally different in that the five-sided cells significantly predominated over the seven-sided cells, which makes the distribution strongly skewed to the right (Fig. 7a). This also concerned both the root proper (Fig. 7b) and specific analyzed tissues (Fig. 7c). However, in the columella and in the lateral regions of the cap, five-sided cells predominated, which caused the distribution to be even more skewed (Fig. 7b). Interestingly, the distribution of the topology of the cells in the computer-generated cell pattern that was obtained by Abera et al. (2014, Fig. 5 ibid) was very similar to the distribution for the radish root apex from our study (Fig. 7). This suggests that the model fits our empirical data well.

### Application of Lewis’s Law

Lewis’s Law was formulated basing on empirical observations (Lewis 1928) and its existence is rather intuitive (Kim et al. 2014). Its applicability as well as dominance of the six-sided cells may be explained by best cell packing, which in further perspective, may be related to best mechanical stress distribution within the living tissue. Let us consider the case of plant cells growing isotropically. Such a growth means that a structure enlarges its dimension, but preserves its shape. A free plant cell with no attachment to other cells would have a shape of a ball mainly because of the turgor pressure. Isotropic growth of such a cell would lead to increase of the ball radius. On the other hand, a plant cell within the tissue consisting of cells of similar type has a shape of a polyhedron whose side number and size are determined by the same traits of the neighboring cells. As plant tissue grows in a symplastic way, any increase of the cell size results in increase of the sizes of its neighbors and the cell division results in a local reorganization of the number of neighbors. Now, let us take into consideration a group of plant cells growing isotropically in which a central non-dividing cell is surrounded by dividing cells. A consequence of a division of the surrounding cell would be an increase of the number of sides of the central cell. Also, a division of each surrounding cell would result in formation of two daughter cells whose number of sides would be smaller than the number of sides of the mother cell. To sum up, all the cells grow isotropically and in a highly coordinated (symplastic) way, the surrounding cells divide automatically causing an increase of the number of contacts of the non-dividing cell (that also have grown) and decrease of the number of contacts of the smaller daughter cells. It is worth mentioning that in case of the dividing cells, the structure preserving the shape is not a single cell, but the complex of cells coming from one mother cell.

Our results show that the linear correlation between the cell area and the number of cell neighbors is present in both the external and internal tissues of the radish root apex, which is an actual plant organ that grows anisotropically. Similar conclusions were drawn in relation to the developed plant cell complexes that were obtained in the above-mentioned computer simulations (Abera et al. 2014) that resulted from the application of the algorithm of cell division. This algorithm assumes several variants of growth anisotropy and cell divisions, namely, various combinations of isotropic/anisotropic growth with symmetric/asymmetric cell division. Interestingly, in each variant of the modeling, Lewis’s Law was fulfilled in this computer-generated cell complex. Although in our study the symmetry/asymmetry of cell divisions was not estimated, it was shown in a modeling study on the growth of the radish root apex (in which the growth field that is shown in Fig. 1 Supplementary Material was used) that the most realistic cell pattern was observed in the case of the symmetric or slightly asymmetric cell divisions that occur along the PDGs (Nakielski 2008). Both the results presented in this paper and the results of Abera et al. (2014) suggest the applicability of Lewis’s Law in plant cells. However, it is important to indicate that the value of the anisotropy coefficient can be correlated with the *r*_L_ value, as was shown here for the radish root apex (Fig. 9). In other words, the largest anisotropy of the growth rate was observed in the region where Lewis’s Law was less manifested. Indeed, the distribution of anisotropy (Fig. 4b) showed a low level of anisotropy in the zones with a high correlation coefficient *r*_L_ from Lewis’s Law (Table 1), namely, in the cc and lc and strong anisotropy in the rp where the coefficient *r*_L_ was lower. Moreover, in the localized in the root proper, ground tissue growth anisotropy appears strongest (Fig. 4c) and *r*_L_ appears lowest (Table 1) of all regions.

A question arises, what is a possible reason for a weaker linear correlation in anisotropically growing plant tissue. In regions of anisotropy, the strongest increase of a cell size takes place along the direction of the strongest tissue growth. In roots, a good example is the elongation zone where the cells elongate in the direction parallel to the root axis, yet they divide rarely. This leads to enlargement of the cell area, but the number of sides increases slower due to rare divisions, which directly affect the lower correlation between the two analyzed traits. In different tissues, the rate of elongation and the rate of cell divisions is different, that is why application of Lewis’s Law on the borders between the tissues may be difficult to interpret. It is worth mentioning that a slight deviation from the linear character of Lewis’s Law was also reported in cucumber epidermis whose cells differed in shape and size from one another (Kim et al. 2014). In this light, anisotropy of either cell growth or of cell shape may be a general cause of loss of correlation between the cell size and the number of its size.

Based on our results, we can state that the growth rate anisotropy factor appears to be a good tool to verify the functioning of Lewis’s Law. Thus, having even fragmentary data about the anisotropy of the growth rates in an organ, we can draw conclusions about the topology of its cell pattern and, what is even more important, vice versa.

The above-presented results provide a new approach to study both the topology and anisotropy of growth in living plant tissues. The applied method enabled the applicability of Lewis’s Law to an anisotropically growing plant organ whose zones grow at different rates to be proven. The proposed tools can be used to validate anisotropic growth via topologic considerations, namely, in case when no in vivo data are available, we can draw a conclusion about level of growth anisotropy of tissue from topological data. Moreover, from the point of view of a modeling study of the growth of plant meristems, the tools may be useful in specification algorithms for the cellular divisions.

## Electronic supplementary material

ESM 1 (PNG 155 kb)

ESM 2 (DOCX 20 kb)

## Abbreviations

GT: Growth tensor
R: Growth rates
PDG: Principal direction of growth

## Zones of the root apex

QC: Quiescent center
rp: Root proper
cc: Columella of the root cap
lc: Lateral regions of the root cap

## Tissues of the root apex

E: Epidermis
G: Ground tissue comprising the cortex and endodermis
S: Stele comprising the pericycle and vascular tissues
*r*_L_: Correlation coefficient between the number of neighboring cells and the cell area
*A*: Anisotropy coefficient
*r*_LA_: Correlation coefficient between *r*_L_ and *A*
