# Supplementary figures and images for: Topological traits of a cellular pattern versus growth rate anisotropy in radish roots
Source: Protoplasma. 2019 Mar 5;256(4):1037–49. doi: 10.1007/s00709-019-01362-6 (PMC6579784; doi:10.1007/s00709-019-01362-6)

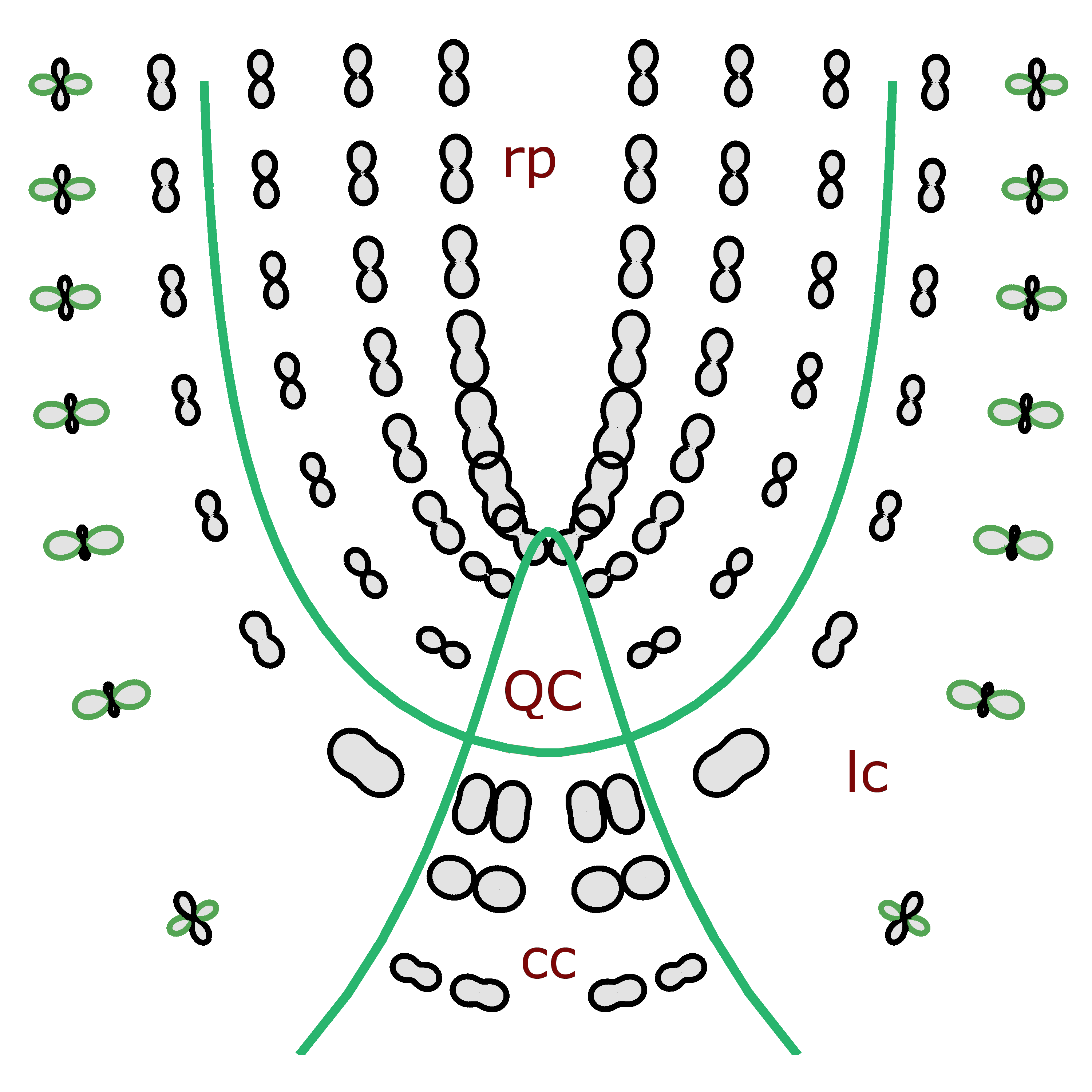

Supplement: Supplementary file 1 — (PNG 155 kb) [file 709_2019_1362_MOESM1_ESM.png]
