# Supplementary material for: Topological traits of a cellular pattern versus growth rate anisotropy in radish roots
Source: Protoplasma. 2019 Mar 5;256(4):1037–49. doi: 10.1007/s00709-019-01362-6 (PMC6579784; doi:10.1007/s00709-019-01362-6)
Supplement: Supplementary file 2 — (DOCX 20 kb) [file 709_2019_1362_MOESM2_ESM.docx]

Supplementary Material

Topological traits of a cellular pattern versus growth rate anisotropy in radish roots.

Anna Piekarska-Stachowiak^1^, Joanna Szymanowska-Pułka^1^, Izabela Potocka^2^, Marcin Lipowczan^1*^

^1^ Department of Biophysics and Morphogenesis of Plants, Faculty of Biology and Environmental Protection, University of Silesia, Katowice, Poland

^2^ Laboratory of Microscopic Techniques, Faculty of Biology and Environmental Protection, University of Silesia, Katowice, Poland

^*^ Author for correspondence: Marcin Lipowczan, e-mail: marcin.lipowczan@us.edu.pl

We used the *log cosh* curvilinear coordinate system (see Fig. 2d in Nakielski 2008) in which four zones can be distinguished: “QC”, “rp”, “cc” and “lc” (Supplementary Material Fig. 1).

The displacement velocity that is related to the growth tensor (GT) consists of two components:

*V_1_ = du/dt* and *V_2_ = dv/dt.*

The assumptions for the zones were:

*V_1_ = 0, V_2_ = 0* for “QC”

*V_1_ = c(u – u_0_), V_2_ = 0* for „rp”

*V_1_ = 0, V_2_ = -k sin(qv)* for „cc”

*V_1_ = c(u – u_0_), V_2_ = -k sin(qv)* for „lc”

where

*q = π/v_0_* and *c = 1, d = 0.3*,

and

*u_0_ = 0.15, v_0_ = 1* and *-1* are borders between zones (green lines in Fig. 2a and in Supplementary Material Fig. 1)

The explicit form of the GT is (see also the supplementary information by Nakielski and Lipowczan 2012, 2013; Kucypera et al. 2017):

$$R_{1}=\frac{1}{h_{u}}\left( \frac{\partial}{\partial u}\frac{\partial u}{\partial t}+\frac{1}{h_{v}}\frac{\partial h_{u}}{\partial v}\frac{\partial v}{\partial t} \right),$$

$$R_{2}=\frac{1}{h_{v}}\left( \frac{\partial}{\partial v}\frac{\partial v}{\partial t}+\frac{1}{h_{u}}\frac{\partial h_{v}}{\partial u}\frac{\partial u}{\partial t} \right),$$

where

$$h_{u}=h_{v}=\frac{\sqrt{{sinh}^{2}\left( 2u \right)+{sin}^{2}\left( 2v \right)}}{{cosh}^{2}\left( u \right)-{sin}^{2}\left( v \right)}$$

are the scale factors.

Figure caption

Supplementary Material Fig. 1 The distribution of the growth rates in the axial plane of the root apex. The figures represent projections of specific indicatrices on the axial plane of the radish root apex and therefore only two orthogonal directions (1 and 2 from Fig. 1.) are considered here. The green curves represent the borders between the specific zones of the apex (QC – quiescent center, rp – root proper, cc – columella of the cap, lc – lateral cap). The rates of growth were calculated based on the growth field that was specified for the radish root apex according to Nakielski (2008). The green elements of the indicatrices refer to negative value of growth rates that indicates local shrinking.
